# The trend in exclusive breastfeeding practice and its association with maternal employment in Bangladesh: A multilevel analysis

**DOI:** 10.3389/fpubh.2022.988016

**Published:** 2022-11-25

**Authors:** Foyez Ahmmed, Md. Jamal Hossain, Tasmiah Sad Sutopa, Md. Al-Mamun, Morshed Alam, Md. Rabiul Islam, Rohit Sharma, Md. Moklesur Rahman Sarker, Mohd Fahami Nur Azlina

**Affiliations:** ^1^Department of Statistics, Comilla University, Cumilla, Bangladesh; ^2^Department of Pharmacy, State University of Bangladesh, Dhaka, Bangladesh; ^3^Department of Statistics, University of Dhaka, Dhaka, Bangladesh; ^4^Department of Sociology, Bangabandhu Sheikh Mujibur Rahman Science and Technology University, Gopalganj, Bangladesh; ^5^Institute of Education and Research, Jagannath University, Dhaka, Bangladesh; ^6^Department of Pharmacy, University of Asia Pacific, Dhaka, Bangladesh; ^7^Department of Rasashastra and Bhaishajya Kalpana, Faculty of Ayurveda, Institute of Medical Sciences, Banaras Hindu University, Varanasi, Uttar Pradesh, India; ^8^Department of Pharmacology, Faculty of Medicine, Universiti Kebangsaan Malaysia, Kuala Lumpur, Malaysia

**Keywords:** exclusive breastfeeding (EBF), maternal employment, Bangladesh demographic and health surveys, early initiation of breastfeeding, multilevel logistic regression

## Abstract

Exclusive breastfeeding (EBF) is essential for infant and child health. This study aimed to explore the trend in the EBF over the last decade in Bangladesh and investigated if there was a significant association with maternal employment by analyzing the data extracted from three consecutive nationally representative surveys: Bangladesh Demographic and Health Surveys (BDHS) of 2011, 2014, and 2017–2018. Prevalence of EBF (95% confidence interval) with the Cochran-Armitage test was reported to see the trend in EBF. A chi-square (χ^2^) test was applied to find the potential factors associated with EBF. Finally, a three-level logistic regression was utilized to find the significant association between maternal employment and EBF while adjusting other covariates. We observed no increase in the practice of EBF over the last decade (*P* = 0.632). The prevalence of EBF was 64.9% (95% CI: 61.41, 68.18) in 2011, followed by 60.1% (95% CI: 56.25, 64) in 2014, and 64.9% (95% CI: 61.82, 67.91) in 2017. Regression results showed that employed mothers had 24% (*p* < 0.05) lower odds of EBF than unemployed mothers. Early initiation of breastfeeding was also found to be significantly associated [Adjusted odds ratio (AOR): 1.22, *P* < 0.05] with EBF. Government and policymakers must come forward with new interventions to increase the practice of EBF, providing basic education and campaigns on the topic of EBF. Maternity leave should be extended up to 6 months of the child's age to achieve an optimal level of EBF.

## Introduction

The term “exclusive breastfeeding” (EBF) refers to feeding newborns only breast milk, not even water, during the first 6 months of life, except for vitamins, mineral supplements, or medications (1). Proper breastfeeding practices are essential for the health and well-being of infants because breast milk offers the necessary nutrients in adequate amounts that may be easily digested and further protect the child against several diseases (2, 3). Hence, the World Health Organization (WHO) prescribes 6 months of EBF, which include just breast milk and no other food or drink, not even water (4). Among children under the age of five, adequate EBF coverage has been projected to prevent between 13 and 15% of deaths (4). EBF recipients were less likely to develop acute respiratory and digestive tract infections than those who did not receive the EBF (5, 6). Exclusively breastfed children have a decreased rate of human immunodeficiency virus (HIV) transmission from their mothers (5). Approximately, 45% of neonatal infectious deaths, 30% of diarrheal deaths, and 18% of acute respiratory infection deaths in children under the age of five are attributed to suboptimal breastfeeding (7). Non-exclusively breastfed infants have a mortality rate of 14.4 times greater than exclusively breastfed infants (6, 8). In addition, EBF also substantially reduces the risk of newborn illness and death and assures healthy early childhood development, including mental and physical growth, by removing the likelihood of contamination from formula milk and other liquids and foods (9). Although EBF can save 13.8% of all fatalities among infants under 2 years and 11.6% of deaths among children under 5 years (10, 11), another study suggested that only 35% of infants were exclusively breastfed globally (11).

Breastfeeding protects against both infectious and non-infectious disorders, including obesity and diabetes, in children (11, 12). To achieve the 2030 Agenda for Sustainable Development, breastfeeding has become a key component in protecting the mother's and child's health (9). Despite the favorable effects of such a natural intervention and the WHO's efforts since 1990, millions of women do not practice EBF. Three out of five infants are not exclusively breastfed leading to an increased risk of death and disease due to not being nursed at an early age (13). Increased EBF practice has slowed in recent years, despite its inclusion as one of six worldwide nutritional goals to be fulfilled by 2025 and as a cornerstone of global maternal and child health agendas (14, 15). Several international organizations are now prioritizing issues and support for funding and developing public-private partnerships to enhance EBF, specifically in low and lower-middle-income countries (LLMICs) (16–19). Breastfeeding for the first 2 years of a child's life and improving nutrition are the main goals of these initiatives (14, 20). As the underlying causes of reduced EBF practice are so complicated, substantial work and ongoing efforts are inevitable. A 6-month EBF practice can be difficult for mothers, particularly in LLMICs, such as Bangladesh, where maternal malnutrition is frequent (16, 19, 21). Lack of knowledge about the advantages of EBF, poor support for mothers at work, and a healthcare system that does not adequately support mothers all contribute to the premature termination of breastfeeding (21). Only 37% of infants under the age of 6 months in poor and middle-income nations are exclusively breastfed (22). For an extended period, lactating mothers in Bangladesh practiced EBF almost exclusively. Bangladesh Demographic and Health Surveys (BDHS) study stated that in 1993–1994 and 1999–2000, EBF prevalence was around 45% (23), 42% in 2004 (24), and 43% in 2007 (25). The prevalence of EBF significantly grew to 64% in the BDHS report in 2011 (26) and decreased to 55% in the BDHS report in 2014 (27). The BDHS 2017 report stated that the current prevalence of EBF hiked to 65% (28).

Several characteristics are linked to how babies are fed worldwide, including income, education, wealth, and work experience (29). EBF has also been hindered by mothers returning to work, especially if the workplace environment is inhospitable for breastfeeding (30, 31). Increasing child malnutrition has been linked to the lack of EBF (32). An essential step toward achieving gender parity is to increase the proportion of women in the workforce in all areas of the economy (33). Supporting the well-being of both children and mothers is a win-win situation (34). As a result of this shift in workplace demographics, it has become increasingly difficult to find solutions to accommodate breastfeeding while simultaneously maintaining one's career (8, 12, 35). EBF practices provide working women with various issues in official and informal sectors (35). Due to the shortness of their maternity leave, they may be less inclined to follow the EBF recommendations if they are forced to return to work (10). Several predictors of EBF and early initiation of breastfeeding (EIBF) in Bangladesh have been explored previously, but nothing is known about the association between the working status of mothers and EBF practice among children in Bangladesh (36, 37). In addition, there is a dearth of complete trend studies of breastfeeding practice and mothers' employment in Bangladesh, along with divisional and regional variations.

The primary purpose of this study was to investigate the trend of EBF practice and determine the association between the EBF status of children and the employment status of mothers using the last three waves of the BDHS data. This study hopes to fill a knowledge gap in Bangladeshi health promotion programs, maternal employment, and public health policy regarding the promotion and enhancement of children's health and well-being through EBF.

## Methodology

### Data

Data for this study was extracted from three consecutive nationally representative surveys: Bangladesh Demographic and Health Surveys (BDHS) of 2011, 2014, and 2017–2018. All the surveys used a list of enumeration areas (EAs)/clusters as a sampling frame constructed by the Bangladesh bureau of statistics (BBS) during the national population census 2011. BDHS used a two-stage stratified cluster sampling procedure. In the first stage, clusters were selected as primary sampling units covering all administrative divisions and places of residence (urban and rural areas). In the second stage, households were selected systematically from each selected cluster. Data from 763 (BDHS survey 2011), 613 (BDHS survey 2014), and 941 (BDHS survey 2017–2018) respondents (15–49 years old women) with their youngest live child living with their mothers and aged 0–5 months were selected for this study. Besides, we eliminated the cases that were missing any of the variables considered in the study. Thus, a total of 2,317 mother–child pairs were used for this study. Further information about the survey is available in the publicly accessible survey reports (26–28).

### Outcome variable

The outcome variable for this study was EBF. Mothers were asked whether they provided their child anything except breast milk during the past 24 h of the interview. Mothers who did not provide any type of food or liquid except breast milk during the last 24 h of the interview were considered exclusively breastfed. Thus, EBF is a binary variable, meaning it is “yes (1)” if a baby is exclusively breastfed and “no (0)” if it is not (26–28).

### Covariates

This study mainly focused on how the practice of EBF changed over the years for employed and unemployed mothers. Mothers who were involved in any economic work in the last 12 months preceding the corresponding surveys were coded as “employed”, otherwise “unemployed”. Additionally, several background characteristics of mothers and children like mothers' current age (≤20, 21–30, >30 years), place of residence (urban, rural), sex of child (male, female), mode of delivery (vaginal, caesarian), mothers' education (no education, primary, secondary, and higher), number of household members (≤5 and >5 persons), birth order (1st, 2nd, 3rd, and ≥4th), number of antenatal care (ANC) visits (<4 times and ≥4 times), facility delivery (‘no' if respondents had a delivery at home, “yes” otherwise), post-natal care (PNC) visits of mothers (“yes” if mother had a PNC within 2 days after delivery, “no” otherwise), and initiation of breastfeeding of the child (“early” if a child put to the breast within 1 h of delivery, “delay” otherwise) were considered.

During the survey years 2011 and 2014, Bangladesh had seven divisions (Dhaka, Chattogram, Khulna, Barishal, Rajshahi, Rangpur, and Sylhet). In 2015, the Dhaka division was divided into two divisions (Dhaka and Mymensingh). Therefore, in the 2017–2018 BDHS report, there were eight divisions. For simplicity of analysis, we merged the Mymensingh division with the Dhaka division for BDHS 2017–2018. Thus, seven geographical areas were considered in the division variable.

Mothers' body-mass index (BMI) is classified into three categories following the definition by the World Health Organization: underweight (<18.5 kg/m^2^), normal (18.5 kg/m^2^ ≤ BMI < 25 kg/m^2^), and overweight (>25 kg/m^2^). Mothers were asked whether they had access to radio or television, or newspapers/magazines. Exposure to media was defined as “yes” if mothers had access to any of the abovementioned media, and “no” otherwise. In BDHS, the wealth index was calculated using principal component analysis based on the available resources of households and divided into five categories (poorest, poor, middle, rich, and richest). For our study, we considered three categories considering the poorest and poor as “poor” and the rich and richest as “rich”.

### Ethical approval

This study used secondary data sets that have been obtained from the BDHS 2011, 2014, and 2017–18 survey data. All participants were informed about the objective of the study, and data were collected after taking consent from each participant. To conduct the surveys, ethical approval was taken by the survey authorities from the National Institute of Population Research and Training (NIPORT) of the Ministry of Health and Family Welfare. We obtained data from the website www.dhsprogram.com upon submitting a request through the website.

### Statistical analysis

This study reported the frequency distribution along with percentages of the background characteristics of children and their mothers considered for analysis by survey year. The prevalence of EBF by the background characteristics in the survey years was also reported through cross-tabulation. The Cochran-Armitage test was used to test the trend in EBF. The pooled data set from three consecutive BDHS data considered in this survey was used for bivariate and multivariate analysis. The Chi-square test was applied to measure the association between covariates and EBF in pooled data (38). Finally, a three-level logistic regression model was applied to observe the adjusted effect of covariates on EBF considering the hierarchical structure of the data. The administrative division was considered a level-3 factor, and the place of residence (urban, rural) was considered a level-2 factor as it is nested into a geographical division.

If *y*_*ijk*_ be the response to EBF for the i'th individual in j'th level-2 (place of residence) and k'th level-3 factors (administrative divisions) with π_*ijk*_ = Pr(*Y*_*ijk*_ = 1), and *x*_1*ijk*_ be the response to a fixed-level covariate for that individual, a three-level logistic random intercept model can be expressed as


(1) $$\begin{array}{l} \text{Level }1:\log\left(\frac{\pi_{ijk}}{1-\pi_{ijk}}\right)=\;\gamma_{0jk}+\;\beta_{1}x_{1ijk} \end{array}$$

(2) $$\begin{array}{l} \text{Level }2:\gamma_{0jk}=\;\gamma_{00k}+\;\beta_{0j0} \end{array}$$

(3) $$\begin{array}{l} \text{Level }3:\gamma_{00k}=\;\beta_{000}+\;\beta_{00k} \end{array}$$

However, to see the cluster effect within the levels taken into consideration in this study, intra-class correlations (ICC) for the null model must be obtained before applying the model, as mentioned earlier. The null model is the regression model where no fixed effect of the covariates is observed. It reveals whether there exists any clustering effect within the clusters through ICC values. That is,


(4) $$\begin{array}{l} \text{Level }1:\log\left(\frac{\pi_{ijk}}{1-\pi_{ijk}}\right)=\;\gamma_{0jk} \end{array}$$

(5) $$\begin{array}{l} \text{Level }2:\gamma_{0jk}=\;\gamma_{00k}+\;\beta_{0j0} \end{array}$$

(6) $$\begin{array}{l} \text{Level }3:\gamma_{00k}=\;\beta_{000}+\;\beta_{00k} \end{array}$$

ICC value lies between 0 and 1, and an ICC value >0 implies the presence of a clustering effect (39, 40). Formulas to obtain ICC values can be written as


(7) $$\begin{array}{l} ICC_{k}=\;\frac{V\left(\beta_{00k}\right)}{V\left(\beta_{0j0}\right)+V\left(\beta_{00k}\right)+\;\left(\frac{\pi^{2}}{3}\right)} \end{array}$$

(8) $$\begin{array}{l} ICC_{J|k}=\;\frac{V\left(\beta_{0j0}\right)+V\left(\beta_{00k}\right)}{V\left(\beta_{0j0}\right)+V\left(\beta_{00k}\right)+\;\left(\frac{\pi^{2}}{3}\right)} \end{array}$$

where, *V*(β_0*j*0_) and *V*(β_00*k*_) are the variances of random intercepts β_0*j*0_ and β_00*k*_ at levels 2 and level 3, respectively.

## Results

### Univariate analysis

**Figure 1:**
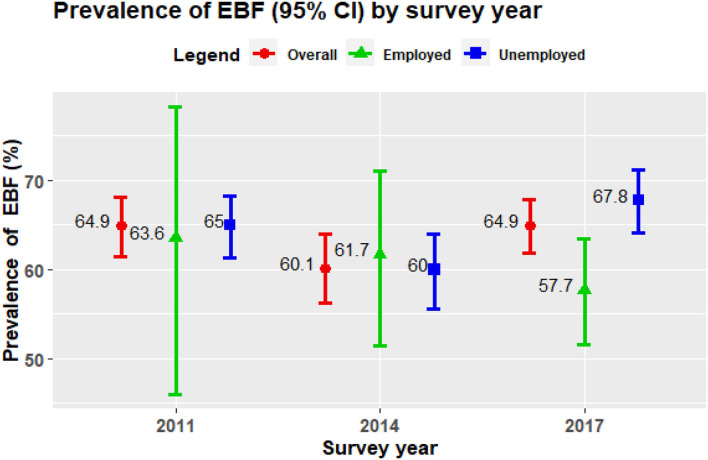
Prevalence of exclusive breastfeeding (EBF) practice (95% confidence interval) among employed and unemployed women in Bangladesh.

Figure 1 shows the prevalence of exclusive breastfeeding in three consecutive BDHS data along with their error bar. The figure represents that ~65% of mothers in BDHS 2017 reported that they exclusively breastfed their child and the prevalence of EBF was previously the same in BDHS 2011. However, the prevalence was lower in BDHS 2014 with only three-fifth (around 60%) of the mothers exclusively breastfeeding their children. Furthermore, the Cochran-Armitage test also showed that there was no trend in the prevalence of EBF (*p* = 0.632).

Table 1 summarizes the percentage distribution of several demographic and socio-economic variables included in this study for the previous three successive BDHS surveys as well as pooled data. Though a major proportion of pooled sample is unemployed (83.0%), an increase of 24.1% of employed mothers was observed from 2011 (4.3%) to 2017 (28.4%). Two-fifth of the respondents in pooled data were residents of the two largest metropolitans of Bangladesh, Dhaka, and Chattogram. Only one-third of the respondents belonged to urban areas. The percentage of households with <5 members increased from 2011 (38.0%) to 2014 (41.7%) was noticeable. Percentage distribution in mothers' education revealed that the proportion of attaining higher education among mothers was rising with time. Most of the mothers in the sample were middle-aged. Despite a substantial drop in underweight mothers, the issue of overweight moms grew with each survey year. However, two-thirds of the mothers in our sample have normal BMIs. The indicators of maternal health care service, such as receiving 4+ ANC (antenatal care), health-facility-based delivery, and receiving postnatal care, have increased by 16, 13.5, and 8.2%, respectively, from 2011 to 2017. However, a considerable proportion of our sample was out of 4+ ANC service and health facility-based delivery. Though birth delivered by C-section has boomed from 2011 (21.1%) to 2017 (33.8%), the majority of birth in our pooled sample was delivered by vaginal delivery (71.9%). The gender ratio (male/female) among children was 1.11 in the pooled sample. Table 1 also shows that more than half of the mothers considered in the pooled sample (52.5%) breastfed their children within 1 h of their birth.

**Table 1:** Percentage distributions of the demographic, socio-economic, and health-related characteristics of the respondents in different survey years as well as for pooled data.

**Variables and**	**Survey year**			**Pooled**
**categories**	**2011** **(*n* = 763) %**	**2014** **(*n* = 613) %**	**2017** **(*n* = 941)**	**(*n* = 2,317) %**
**Maternal employment**				
Unemployed	95.7	84.7	71.6	83.0
Employed	4.3	15.3	28.4	17.0
**Division**				
Barishal	9.2	14.0	11.6	11.4
Chattogram	21.6	19.9	17.2	19.4
Dhaka	16.4	16.5	27.6	21.0
Khulna	14.2	10.9	11.1	12.0
Rajshahi	12.5	11.3	12.2	12.0
Rangpur	12.3	12.2	9.2	11.0
Sylhet	13.9	15.2	11.1	13.1
**Place of residence**				
Urban	32.1	32.0	35.8	33.6
Rural	67.9	68.0	64.2	66.4
**Wealth index**				
Poor	38.4	39.5	41.1	39.8
Middle	20.1	19.4	20.8	20.2
Rich	41.5	41.1	38.0	40.0
**No. of household members**				
≤ 5	38.0	43.9	41.7	41.0
>5	62.0	56.1	58.3	59.0
**Exposure to media**				
No	35.4	37.0	39.2	37.4
Yes	64.6	63.0	60.8	62.6
**Mother's education**				
No education	15.6	12.9	6.8	11.3
Primary	28.0	26.3	28.7	27.8
Secondary	48.0	43.9	45.5	45.9
Higher	8.4	17.0	19.0	15.0
**Mother's current age**				
≤ 20	34.9	37.7	30.1	33.7
21–30	55.4	50.9	56.6	54.7
>30	9.7	11.4	13.3	11.6
**Mother's BMI**				
Under-weight	21.1	18.8	12.2	16.9
Normal	67.5	60.5	68.2	65.9
Overweight	11.4	20.7	19.6	17.2
**No. of ANC received**				
< 4	70.2	66.6	54.2	62.8
≥4	29.8	33.4	45.8	37.2
**Facility delivery**				
No	62.6	53.3	49.1	54.7
Yes	37.4	46.7	50.9	45.3
**Received postnatal care**				
No	42.5	32.5	34.3	36.5
Yes	57.5	67.5	65.7	63.5
**Mode of delivery**				
Vaginal	78.9	71.9	66.2	71.9
Caesarian	21.1	28.1	33.8	28.1
**Sex of child**				
Male	51.6	55.0	52.4	52.8
Female	48.4	45.0	47.6	47.2
**Birth order**				
1st	37.6	44.0	36.7	38.9
2nd	29.4	28.1	32.8	30.4
3rd	17.6	16.3	17.9	17.4
≥4th	15.5	11.6	12.6	13.3
**Initiation of breastfeeding**				
Late	51.8	49.3	42.9	47.5
Early	48.2	50.7	57.1	52.5

### Bivariate analysis

This study tried to find the trends in the practice of EBF, considering different covariates over the last three BDHSs, specifically considering maternal employment status. Figure 1 exhibits the changing pattern in the prevalence of EBF practice among mothers by their employment status from 2011 to 2017. There has been a notable decrease in the prevalence of EBF from 2011 (63.6%) to 2017 (57.7%). Though findings from BDHS 2014 survey suggested that employed mothers have a higher prevalence (61.7%) than unemployed mothers (60%), the results from BDHS 2011 and BDHS 2017 displayed a lower prevalence for employed mothers (63.6 and 57.7%, respectively) than their opposite counterparts (65 and 67.8%, respectively). However, the bivariate analysis in the pooled data, as shown in Table 2, reported that maternal employment was significantly associated with EBF practice, and the prevalence is lower among employed mothers (59.1%) compared to unemployed mothers (64.6%).

**Table 2:** Prevalence of exclusive breastfeeding (EBF) by different background characteristics. *p*-values were generated from the chi-square test of pooled (2011–2017) data.

**Variables**	**Categories**	**2011**	**2014**	**2017**	**Pooled**	***P*-value**
Maternal	Unemployed	64.9	59.9	67.8	64.6	**0.040**
employment	Employed	63.6	61.7	57.7	59.1	
Division	Barishal	52.9	43.0	63.3	54.0	**<0.001**
	Chattogram	69.1	60.7	78.4	70.2	
	Dhaka	55.2	46.5	62.7	57.4	
	khulna	64.8	65.7	53.8	60.9	
	Rajshahi	66.3	56.5	51.3	57.7	
	Rangpur	75.5	78.7	71.3	75.0	
	Sylhet	67.0	74.2	72.1	71.0	
Place of residence	Urban	65.3	57.1	60.5	61.2	**0.078**
	Rural	64.7	61.6	67.4	64.9	
Wealth index	Poor	60.1	61.2	63.6	61.8	0.299
	Middle	66.7	61.3	63.8	64.1	
	Rich	68.5	58.7	67.0	65.3	
No. of Household	≤ 5	67.6	59.5	59.4	61.9	0.150
members	> 5	63.2	60.8	68.9	64.9	
Exposure to media	No	63.3	58.6	66.4	63.4	0.837
	Yes	65.7	61.1	64.0	63.8	
Mothers education	No education	59.7	58.2	64.1	60.3	0.140
	Primary	61.7	57.8	64.4	61.9	
	Secondary	66.1	61.3	64.0	64.1	
	Higher	78.1	62.5	68.2	68.3	
Mothers' current	≤ 20	70.3	54.5	66.1	64.1	0.201
age	21-30	62.2	64.4	66.2	64.4	
	> 30	60.8	60.0	56.8	58.7	
Mothers BMI	Under-weight	66.5	52.2	61.7	60.9	0.180
	Normal	65.8	62.0	66.0	65.0	
	Overweight	56.3	62.2	63.0	61.3	
No. of ANC	< 4	63.2	61.3	65.5	63.5	0.815
received	≥4	68.7	58.0	64.3	64.0	
Facility delivery	No	64.0	59.3	64.3	62.9	0.406
	Yes	66.3	61.2	65.6	64.6	
Received postnatal	No	64.2	63.3	66.9	65.0	0.305
care	Yes	65.4	58.7	63.9	62.9	
Mode of delivery	Vaginal	65.8	61.2	64.8	64.2	0.365
	Caesarian	61.5	57.6	65.1	62.2	
Sex of child	Male	62.9	59.1	63.5	62.1	**0.097**
	Female	66.9	61.6	66.5	65.4	
Birth order	1st	67.2	56.7	66.1	63.6	0.367
	2nd	71.4	62.2	64.1	66.0	
	3rd	56.7	65.0	63.1	61.4	
	≥4th	55.9	62.0	66.4	61.4	
Initiation of	Delay	65.1	56.3	62.6	61.8	**0.071**
breastfeeding	Early	64.7	64.0	66.7	65.4	

Bold mean the level of significance.

While investigating the association between the EBF and the rest of the independent variables, we found that the EBF is significantly associated with administrative divisions. Among these administrative divisions, the percentage of EBF practice was the lowest in Barisal (54.0%), whereas Rangpur had the highest prevalence of EBF practice (75%). Moreover, place of residence also significantly impacted the practice of EBF at a 10% significance level (*p* = 0.078). Mothers in rural areas had a higher prevalence (64.9%) than mothers in urban areas (61.2%). Regarding the child's gender, the male child has a lower prevalence of EBF (62.1%) than the female child (65.4%). However, the children who got breastfeeding within 1 h of birth had a higher prevalence (65.4%) than their counterparts (61.8%).

### Multivariate analysis

Table 3 shows the results obtained from multivariate analysis applied to the pooled data in this study. The covariates found to be significantly associated with EBF in bivariate analysis were included in multivariate analysis. Besides, three covariates (year, wealth index, and mother's current age), which were significantly associated with EBF in previous literature (33, 41, 42), were also considered in the multivariate analysis to explore their adjusted effect on EBF.

**Table 3:** Three-level logistic random intercept model using “place of residence” as level-two and “administrative division” as level-three factors to find the factors associated with exclusive breastfeeding (EBF) practice in Bangladesh.

**Covariates**	**Categories**	**AOR (95% CI)**	***P*-value**
Year	2011	1.00	
	2014	0.82 (0.66, 1.04)	0.101
	2017	1.10 (0.89, 1.37)	0.362
Maternal employment	Unemployed	1.00	
	Employed	0.76 (0.59, 0.96)	**0.027**
Mothers' current age	≤ 20	1.00	
	21–30	1.01 (0.82, 1.21)	0.988
	>30	0.82 (0.61, 1.11)	0.218
Sex of child	Male	1.00	
	Female	1.16 (0.98, 1.38)	**0.091**
Mothers education	No education	1.00	
	Primary	1.07 (0.79, 1.46)	0.633
	Secondary	1.10 (0.81, 1.49)	0.514
	Higher	1.41 (0.98, 2.06)	**0.068**
Wealth index	Poor	1.00	
	Middle	1.04 (0.81, 1.33)	0.727
	Rich	1.13 (0.90, 1.43)	0.279
Initiation of Breastfeeding	Late	1.00	
	Early	1.22 (1.03, 1.45)	**0.025**
Constant		0.816 (0.90, 2.03)	0.316
AIC	3004.401		
**Random-effect parameters**	**Var (Place of residence)**	**Var (Division)**	
Coefficients (95% CI)	0.031(0.003,0.259)	0.084(0.018,0.389)	
Intra-class correlation coefficient (ICC)	0.033	0.024	
Null model			
Constant		1.77	<0.001
Coefficients (95% C.I)	0.018 (0.001,0.292)	0.082 (0.019,0.346)	
Intra-class correlation coefficient (ICC)	0.029	0.024	
AIC	3009.182		

Bold mean the level of significance.

Regarding mothers' employment status, employed mothers had 24% lower odds of EBF practice than unemployed mothers (AOR = 0.76), and the covariate (maternal employment) was significantly associated with EBF practice (*p* = 0.027; Table 3). Furthermore, Table 3 reveals that despite having lower odds ratio in 2014 (AOR = 0.82) compared to 2011, the odds ratio of EBF practice became higher in 2017 (AOR = 1.10) relative to 2011. However, the adjusted effect of the survey year on EBF practice was insignificant, indicating no change in EBF prevalence with the progression of time from 2011 to 2017.

Moreover, female children had 16% higher odds of being exclusively breastfed than male children (AOR = 1.16, 95% CI = 0.98, 1.38, *p* = 0.091). The highly educated mothers had 41% higher odds of EBF practice than mothers without education. The mothers with primary and secondary education levels had 7% (AOR = 1.07, *p* = 0.633) and 10% (AOR = 1.10, *p* = 0.514) higher odds of EBF practice, respectively, than mothers without education. The mothers from the higher education category had 41% higher odds than the mothers from the no education group (AOR = 1.41, 95% CI = 0.98, 2.06, *p* = 0.068). The mothers who initiated breastfeeding early after the birth of their child have 22% higher odds of EBF practice (AOR = 1.22) than those who initiated breastfeeding later.

At first, a null model without any covariates was applied to the pulled dataset to justify using the three-level multilevel logistic regression model. Table 3 illustrates that the estimated variance for a random intercept from the null model was 0.018 for a place of residence and 0.082 for administrative divisions, respectively. The 95% confidence interval (95% CI) of the estimated variance, as mentioned in Table 3, clearly indicated that both estimates were significantly different from 0, which means considerable heterogeneity exists in EBF practice concerning the administrative divisions and places of residence within the administrative divisions when no other covariates are considered in the model. The intra-class correlation coefficient (ICC) for the place of residence was reported as 0.029, which indicated that 2.9% of the total variation in EBF practice among mothers could be explained by variation in place of residence. Similarly, the ICC for the division was 0.024, which implied that 2.4% of the total variation in EBF practice among mothers was accountable to variation in place of residence. As the null model justified the presence of random effect in place of residence and division level, further multivariate analysis in this study proceeded with a three-level multilevel analysis, constructing a full model, including all other covariates.

## Discussion

Exclusive breastfeeding practice can be regarded as an appropriate strategy to enhance both child survival and maternal health. To ensure a balanced diet and reduce infant mortality and morbidity, exclusively breastfeeding newborns for the first 6 months of their life is crucial (18, 43). In this study, using the previous three waves of the BDHS data, we have conducted a multilevel analysis to find out the trend of EBF practices and determine the association between children's EBF practice and mothers' job status.

The current research's findings have shown the trends in EBF and its association with maternal employment status in Bangladesh, along with cluster variation at the divisional and regional levels. The study mainly found that the newborns of working mothers are more deprived of EBF practice than their counterparts. The study also found a cluster effect, including administrative divisions and place of residence, mainly indicating regional variation at those levels.

It is evident from the current study that slightly more than two-thirds of mothers exclusively breastfed their children (Figure 1). These findings supported several prior studies conducted in Bangladesh (41, 44). However, this EBF rate has significant differences from some other countries. The prevalence of EBF in Bangladesh was higher than in certain other countries, including India (Tamil Nadu, 34%), Saudi Arabia (Al-Hassa, 24.4%), the United States (16.8%), and Malaysia (Peninsular, 43.1%) (45–48). Socioeconomic and cultural factors may be behind the lower EBF rates in other countries. This rate may also vary due to methodological differences in research. The current analysis also found that more than half of the mothers breastfed children immediately after their birth. Almost similar data have been found in another study conducted by Ekubay et al. in Ethiopia (49). UNICEF and WHO also suggest breastfeeding exclusively during the first 6 months of life, beginning immediately after birth (50).

The study found that maternal employment was significantly associated with EBF practice and the prevalence was lower among employed mothers than unemployed mothers. Some previous studies have also found similar results (18, 51). One of the reasons behind the low EBF rate of educated mothers may be that they do not have enough time to breastfeed because of their job workload (52). In many cases, children are raised by midwives and as a result, children of educated mothers are deprived of breastfeeding (53). As a result, educated and employed mothers may be unable to manage enough breastfeeding time during working hours. However, such deprivation of EBF toward newborns of working mothers is tried to be mitigated to some extent. Although working mothers in Bangladesh who are in government service get 6 months of maternity leave, it is not offered in most private-sector jobs (54). There is also a lack of a suitable environment (such as a breastfeeding zone) in the workplace. That is why mothers feel shy to breastfeed their children at the workplace (55). Thus, the immense workload, unavailability of maternal leave, and scarcity of suitable space for breastfeeding may compel working mothers to refrain from practicing EBF.

Apart from examining the general prevalence of EBF in Bangladesh, this study examined the cluster effect of EBF practice among mothers in representative regions. This study found that the respondent's location (administrative division) was significantly associated with exclusive breastfeeding. The rate of breastfeeding was higher in the Rangpur region. The relationship between location and breastfeeding was also seen in other studies (18). The study also manifested the existence of a cluster effect at the divisional level and the level of the type of residence, which is supported by previous studies (41). The mothers residing in the same cluster, in general, receive a similar variety of maternal health services. Moreover, they have the same background characteristics, such as educational status, household wealth index, etc., which may differ among clusters. This could explain the cluster effect among administrative divisions and the urban–rural intra-cluster correlation revealed in this study (18, 19).

Our findings revealed that almost half of the mothers breastfed their children within 1 h of their birth. According to Lawn et al., about 4 million newborns die every year globally due to the absence of the mother's breast immediately after birth, which suggests that there is no substitute for breast milk to reduce infant mortality (56). The current analysis also found that nearly two-thirds of adolescent mothers exclusively breastfed. On the other hand, several previous studies were exceptions to our study. A Brazilian study indicated that adolescent mothers had a lower rate of EBF than older mothers (57). A Chinese study also found that teens are more likely to be embarrassed to breastfeed their babies (58). Many researchers believe that biological, socio-cultural, personal, and environmental factors play a role in breastfeeding babies (59, 60). A study conducted in Rajshahi and Pabna (two districts in Rajshahi Division, Bangladesh) showed that mothers with a higher socioeconomic status frequently experience shyness or embarrassment when breastfeeding in public places (61). Similar notions have been found in other studies (62). For example, in some parts of Africa, the rate of exclusive breastfeeding is very low because African cultures regard the female breast as an integral element of a woman's identity and femininity that must be kept private (63).

Our analysis also found that the early initiation of breastfeeding category showed 22% more EBF practice than the late initiation of breastfeeding category. Within an hour of birth, breastfeeding should be started to give the newborn the essential nutrients together with colostrum (first milk). Colostrum strengthens the newborn's immune system, growth hormones, and other protective elements. Compared to mothers who breastfed their babies within an hour of birth, those who did not breastfeed their babies had a greater chance of neonatal death (64, 65). According to research by Ahmmed et al., EIBF was less likely to occur in mothers who had caesarian deliveries, gave birth in private facilities, had multiple babies, or were older at the time of delivery (37). Furthermore, mothers' education (*p* = 0.068) and the gender of children (*p* = 0.091) were also associated with EBF practice at a 10% significance level. However, several previous studies found no significant association between these factors with EBF practice (17, 18). Furthermore, the current multivariate analysis showed no significant association between the wealth index of mothers with EBF rates. Similarly, the study by Joshi et al. found no significant association between mothers' wealth index and EBF rates in Bangladesh (18).

There are some limitations to this study. Because of the retrospective nature of the study, the findings may be subject to recall bias. We used an unweighted sample which may not be useful in providing the point prevalence of EBF. We can extend our study considering sampling weight as further scope. Since the data were cross-sectional, we were not allowed to evaluate the temporal direction of the effects. Furthermore, some studies revealed that traditional belief was significantly associated with EBF. But we could not include this in our study as this variable was not available in BDHS data.

### Policy implication and future research

Further study about EBF practice among mothers is needed to establish policies that will increase breastfeeding rates and enable the WHO to achieve its objective of 90% EBF at 6 months (66). For all types of public-private jobs, mothers should have at least 1 year of maternity leave. Breastfeeding zones need to be established for mothers in the workplace. There should be daycare facilities for children in the mother's workplace. EBF practice in Bangladesh could increase by adopting relevant labor laws pertaining to maternal care in both the public and private sectors. Additionally, ethnographic research is required to better understand the social and familial factors that may encourage EBF. Additional treatments could include using mass media, targeted home visits by health surveillance assistants, and increasing male involvement by emphasizing the necessity of EBF and offering support to partners to sustain it. In such studies, it is often impossible to get in-depth information with statistical data. In that case, some qualitative research can be conducted. To improve good health for children and achieve SDG 3, which is to reduce neonatal death by 2030 globally, communities and health facilities must be strengthened as soon as possible.

## Conclusion

The current study assessed the trend in the EBF practice by analyzing the 3 years of the most recent BDHS survey data (2011, 2014, and 2017–2018) in Bangladesh and examined if there was a significant association between maternal employment and the practice of EBF. Unemployed women significantly exerted a higher chance of EBF practice. There was no significant increase in the prevalence of EBF in Bangladesh in the three BDHS survey data. Several steps should be taken immediately to increase the EBF practice at this current stage. Encouragement of EBF practice should be the highest priority among all sectors of society. It is necessary to provide an appropriate baby-friendly workplace for breastfeeding mothers to continue their EBF practice. Furthermore, implementing relevant labor acts regarding maternity care in both the public and private sectors may significantly increase EBF practice in Bangladesh. EBF practice would be increased through outreach programs to ensure increased healthcare service utilization during pregnancy and delivery, and individual and community-level awareness of EBF practice.

## Data availability statement

Publicly available datasets were analyzed in this study. This data can be found here: www.dhsprogram.com.

## Ethics statement

The studies involving human participants were reviewed and approved by National Institute of Population Research and Training (NIPORT) of the Ministry of Health and Family Welfare, Bangladesh. The patients/participants provided their written informed consent to participate in this study.

## Author contributions

FA, MH, and TS developed the idea of the work and designed the study. FA and TS collected the data and cured and analyzed the raw data. FA, MH, TS, MA-M, and MAl interpreted the analyzed data, searched the literature, and drafted the original manuscript. MH, MS, and MAz have made funding acquisitions. MI, RS, MS, and MAz critically revised and improved the manuscript. All authors reviewed and approved the final version of the manuscript.

## Conflict of interest

The authors declare that the research was conducted in the absence of any commercial or financial relationships that could be construed as a potential conflict of interest.

## Publisher's note

All claims expressed in this article are solely those of the authors and do not necessarily represent those of their affiliated organizations, or those of the publisher, the editors and the reviewers. Any product that may be evaluated in this article, or claim that may be made by its manufacturer, is not guaranteed or endorsed by the publisher.
